# The Optimal Management of Acute Febrile Encephalopathy in the Aged Patient: A Systematic Review

**DOI:** 10.1155/2016/5273651

**Published:** 2016-02-17

**Authors:** Fereshte Sheybani, HamidReza Naderi, Sareh Sajjadi

**Affiliations:** Department of Infectious Diseases, Faculty of Medicine, Mashhad University of Medical Sciences, Mashhad 91388-13944, Iran

## Abstract

The elderly comprise less than 13 percent of world population. Nonetheless, they represent nearly half of all hospitalized adults. Acute change in mental status from baseline is commonly seen among the elderly even when the main process does not involve the central nervous system. The term “geriatric syndrome” is used to capture those clinical conditions in older people that do not fit into discrete disease categories, including delirium, falls, frailty, dizziness, syncope, and urinary incontinence. Despite the growing number of elderly population, especially those who require hospitalization and the high burden of common infections accompanied by encephalopathy among them, there are several unresolved questions regarding the optimal management they deserve. The questions posed in this systematic review concern the need to rule out CNS infection in all elderly patients presented with fever and altered mental status in the routine management of febrile encephalopathy. In doing so, we sought to identify all potentially relevant articles using searches of web-based databases with no language restriction. Finally, we reviewed 93 research articles that were relevant to each part of our study. No prospective study was found to address how should AFE in the aged be optimally managed.

## 1. Background

The world population has been experiencing significant aging. The result of this trend is the rising proportions of older persons in the total population that increased from 9.2 percent in 1990 to 11.7 percent in 2013 and will continue to grow as a proportion of the world population, reaching 21.1 percent by 2050. It has been estimated that older persons exceed the number of children for the first time in 2047 [1]. Although patients 65 years and older comprise less than 13 percent of the population, they represent 40 percent of hospitalized adults and nearly half of all healthcare dollars spent on hospitalization [2]. It has been estimated that annual healthcare costs for the elderly are approximately four to five times those of people in their early teens [3]. With the continuing trend of population aging, hospitalization and healthcare spending for older adults are expected to be rising [2]. Despite the disproportionate prevalence of hospitalized patients who are in the older age range, hospitalist programs often do not emphasize the need for geriatric skills and most hospital-based clinicians are not trained to treat older adult patients [2]. The elderly people require special care and attention when acutely ill. There are several distinct conditions related to elderly population. The term “geriatric syndrome” is used to capture those clinical conditions in older persons that do not fit into discrete disease categories, including delirium, falls, frailty, dizziness, syncope, and urinary incontinence. Although the concept of the geriatric syndrome remains poorly defined, four underlying risk factors have been identified for this syndrome, including older age, cognitive impairment, functional impairment, and impaired mobility [4]. When an older adult with several chronic medical conditions develops an acute illness, one or more organ systems may fail. In addition, because of age-related diminution of physiologic reserves and greater vulnerability of elderly to acute stress, other organ systems that are seemingly unrelated to the presenting problem may lack the reserve to withstand the stresses of the acute illness. As a result, patient presents with failure of the organs that appear apart from the original complaint for which the patient is hospitalized [2]. Therefore, the chief complaint frequently does not represent the specific pathologic condition underlying the change in health status [5]. In some cases, the two processes may involve distinct and distant organs with a disconnect between the site of the underlying physiologic insult and the resulting clinical symptom. The fact that these syndromes cross organ systems along with their multifactorial nature challenges traditional ways of viewing clinical care and research [4]. For example, changes in mental status from baseline are commonly seen even when the main process does not involve the central nervous system (CNS) [6]; this is the consequence of altered neural function in the form of cognitive and behavioral changes which permits the diagnosis of delirium [4]. Delirium is one of the most frequent presentations in the elderly that result from diminished cognitive reserve and brain dysfunction [7]. It is an under recognized public health problem that affects significant number of older emergency department (ED) patients. With the elderly population expected to grow exponentially over the next several decades, delirium's burden on EDs will intensify. Delirium is defined as an acute change in cognition that cannot be better accounted for by preexisting or evolving dementia. This change in cognition is rapid, occurring over a period of hours or days, and is classically described as reversible [8]. Delirium in hospitalized patients is most closely associated with factors already present on admission such as prior cognitive impairment and advanced age [7]. The incidence of delirium increases progressively after the forth decade of life [9]. In elderly patients it usually manifests as confusion or altered mental status. Although confusion can be a presentation of dementia or psychological disorders, until other causes of confusion are identified, the confused patient should be assumed to have delirium, which is often reversible with treatment of the underlying disorder [9]. Delirium may result from a number of general medical and neurologic conditions; the most common causes are medical conditions such as infections. Aging causes the increased susceptibility to infection that is most likely a reflection of the age-associated decline in the competence of the immune system. Moreover, both morbidity and mortality from any infections may be severalfold higher in the elderly [10, 11]. Infection is the primary cause of death in one-third of individuals aged 65 years and older and is a contributor to death for many others [12]. A change in mental status or decline in function may be the only presenting problem in an older patient with an infection [13]. In this situation one should distinguish between infections, especially those involving CNS, versus noninfectious causes of altered mental status.

In fact, one of the common problems encountered by the physicians in ED is identification of cause and treatment of patients who present with acute onset of fever and altered mental status, not only to ensure survival but also to prevent long-term neurological sequelae. This is the fact especially in the management of older adult patients with acute febrile encephalopathy (AFE) and several chronic medical conditions. AFE is a common presentation among older adults who admitted to the ED. AFE is used to describe patients with a condition in which altered mental status either accompanies or follows a short febrile illness [14]. Recently, it has been suggested that the term “acute encephalitis syndrome” (AES) be used instead of AFE. A case of AES is defined as a person of any age, at any time of year, with the acute onset of fever and a change in mental status and/or new onset of seizure (excluding simple febrile seizures) [15]. Although the clinical definition of AES was introduced to facilitate surveillance for Japanese Encephalitis, this definition is broad and includes illnesses caused by many infectious as well as noninfectious etiologies [16]. Presentation with encephalopathy following short febrile illness is more common among the elderly. Considering the fact that confusion (altered mental state) is the hallmark of encephalopathy, it would be present in up to 50 percent of elderly hospitalized patients, 10 percent of all hospitalized patients, and 2 percent of ED patients [17]. According to Han et al. 7 to 10 percent of older ED patients are delirious [8]. It has been noted that CNS infections are the most common causes of altered mental status in patients with nontraumatic coma [14]. Here, a question comes to mind: is it true for elderly patients who present with altered mental status to the ED? Many physicians face great number of febrile encephalopathic older adults in the ED most of whom do not have CNS infections. In fact, although one of the most important differential diagnoses in a confused elderly, who present to ED, especially when altered mental status is accompanied with fever or sepsis syndrome, is CNS infection, this type of infection does not seem to be the most common underlying cause. In this regard, is it reasonable to perform lumbar puncture (LP) on all febrile older patients who admitted with acute alteration in mental status after normal neuroimaging results?

Despite the growing number of elderly population, especially those who require hospitalization as well as the high burden of altered mental status among older ED patients, there are several unresolved questions about the optimal management of elderly patients with AFE that require hospitalization. Therefore, we decided to answer these questions by searching in the literature in the form of a systematic review. The questions posed in this review concern the need of early LP in all AFE elderly patients in the routine management of AFE. The questions are as follows:

1. What is the etiologic distribution of AFE in elderly patients?
2. Is higher prevalence of CNS infection among the elderly responsible for higher frequency of AFE in this age group? What is the real frequency of CNS infection among elderly patients who present with AFE?
3. Does sepsis-associated encephalopathy (SAE) explain the higher prevalence of AFE among elderly patients that require hospitalization?
4. Is the classic triad of meningitis a sensitive indicator of CNS infection among elderly patients? Is the combination of nuchal rigidity and encephalopathy a specific indicator of CNS infection in elderly patients with AFE?
5. Is a LP necessary when evaluating an older patient with AFE? How many elderly patients with AFE undergo LP? Too many or too few?

Finally, we discuss the necessity for developing an evidence-based guideline for optimal management of AFE in the elderly.

## 2. Methods

### 2.1. Search Strategy

We sought to identify all potentially relevant articles using searches of web-based databases (Google Scholar, Medline, PubMed, Scopus, and ResearchGate) with no language restriction. Search terms were “elderly,” “geriatric,” “aged adult,” “older adult,” “confusion,” “delirium,” “febrile,” “fever,” “encephalopathy,” “altered mental status,” “acute encephalitis syndrome,” “febrile encephalopathy,” “lumbar puncture,” and “LP.” Potentially relevant studies were retrieved and reviewed by 2 reviewers. Disagreements were resolved by discussion between the two review authors; if no agreement could be reached, it was planned that a third author would decide. The references of the retrieved articles were examined for pertinent studies.

### 2.2. Study Selection

**Figure 1:**
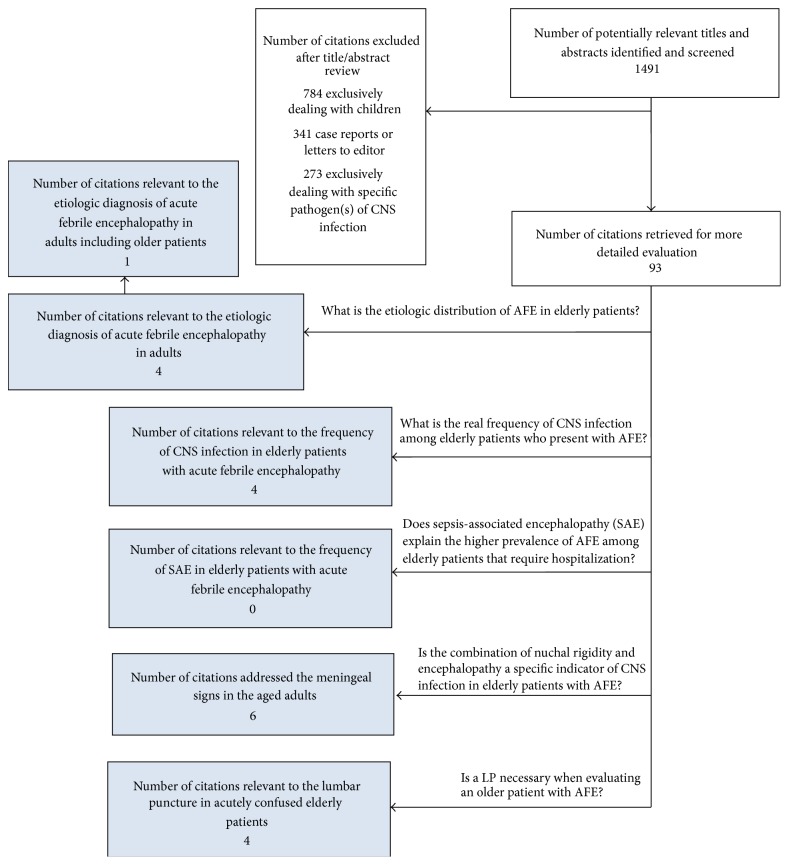
Relevant studies identified from the literature search. CNS: central nervous system; LP: lumbar puncture; SAE: sepsis-associated encephalopathy; and AFE: acute febrile encephalopathy.

By reviewing the titles of articles, we identified 1491 candidate articles for inclusion in our literature review. Of these 1398 were eliminated after review of their abstracts. The reasons for exclusion were as follows: 784 because they were related to children, 341 because they were case reports or letter to editors, and 273 because they exclusively dealt with a specific pathogen(s) of CNS infection in elderly. The remaining 93 articles underwent full text review, which eliminated another 79 that were not relevant to the study questions. Out of the finally selected articles, 4 were relevant to lumbar puncture and frequency of CNS infection in older patients with encephalopathy, 6 addressed meningeal signs in elderly, and 4 were relevant to etiologic diagnosis of AFE in adults (Figure 1).

#### 2.2.1. First Question: What Is the Etiologic Distribution of Acute Febrile Encephalopathy (AFE) in Elderly Patients?

Virtually every medical condition is capable of causing confusion. The most frequent disorders causing altered mental status are common systemic disorders, such as urinary tract infections or pneumonia. The elderly, particularly those with some chronic cognitive impairment, are the most vulnerable group. Patients with dementia who develop a systemic illness can present with an acute change in mental status. The first challenge facing the emergency clinician is to define what is meant by altered mental status or confusion and to ascertain why it led to the emergency department (ED) visit [17]. When confusion is accompanied by fever or sepsis syndrome, the possibility of infection especially central nervous system (CNS) infection as the etiologic cause of alteration in mental status comes to mind. In this part, we try to determine which underlying etiologies are more frequently presented as AFE in elderly patients admitted to the ED.

*Systematic Review*. There are numerous studies performed to address the etiology of AFE in children; however, we found only 4 studies in the literature that evaluated the etiologic diagnosis of AFE in adult patients [14, 22, 27, 28]. None of them make comparison between younger adults and the elderly. One out of 4 studies excluded patients older than 60 years [28]. The mean age of participants of the 3 other studies was between 30 and 40 years. Therefore, older adults had not a significant role in these studies. In 3 of these studies the most frequent etiologic diagnoses of AFE in adult patients were pyogenic meningitis, viral encephalitis, and sepsis-associated encephalopathy (SAE), and less frequently tuberculous meningitis (TM), cerebral malaria, leptospirosis, and brain abscess.

In forth study that included patients with acute encephalitis syndrome (fever, headache, altered mental status, vomiting, seizure, and neurodeficit), 25 (13.1 percent) out of 190 patients were elderly cases. The results of this study showed 99 (52 percent) patients with meningitis including 7 patients with confirmed bacterial meningitis (BM) and 13 with CSF neutrophilic pleocytosis. Others finally were diagnosed as acute hepatic encephalopathy, metabolic encephalopathy, alcoholic encephalopathy, cerebral malaria, brain abscess, SAE, and so forth [22].

The profile of febrile encephalopathy varies across different geographic areas. There would be substantial variation in the distribution of etiologic diagnosis of AFE among different studies on the basis of the age range of the participants, as well as the population studied. All of the above-mentioned studies were performed in India that is a tropical area with high endemicity for tuberculosis and malaria. Thus, the results of these studies cannot be generalized to other population.

The conclusion is that there is no enough information about the etiologic distribution of AFE in adults including elderly population except few studies from limited parts of the world.

#### 2.2.2. Second Question: Is Higher Prevalence of Central Nervous System (CNS) Infection among the Elderly Responsible for Higher Frequency of Acute Febrile Encephalopathy (AFE) in This Age Group? What Is the Real Frequency of CNS Infection among Elderly Patients Who Present with AFE?

Bacterial meningitis (BM) remains one of the most feared infectious diseases because of its subtle onset and high mortality rate. Although the incidence of meningitis is the highest among infants during the first month of life, several large studies have documented a later peak of incidence among persons aged 60 and over. Estimates of the incidence of meningitis in these patients range from 2 to 9 per 10^5^ per year [10]. In recent years, BM has radically changed to become a disease largely of adults—in particular, of older adults [29]. The introduction of conjugate vaccines and preventive treatment of colonized pregnant women have had a major impact on the epidemiology and characteristics of BM [30]. This circumstance highlights key problem areas in its management including recognition of the disease in older patients who present with fewer of the classic symptoms of meningitis or for whom there are other explanations for these symptoms [29]. In the prospective study conducted by Domingo et al. on 635 episodes of acute bacterial meningitis (ABM) in adult patients in Barcelona, the corresponding incidence was 4.03 per 10^5^ and 7.40 per 10^5^ inhabitants/year for patients aged 15 to 64 years and patients aged ≥ 65 years, respectively [31]. The higher prevalence of meningitis among the elderly compared with the general adult population has been also documented in developing countries [32]. Although older age is a known risk factor for CNS infection, estimation of the true prevalence of CNS infection in febrile aged patients with acute alteration of mental status has received little attention. In the study conducted by Cagatay et al. 25 out of 135 (18.5 percent) acutely febrile aged patients with final diagnosis of infectious disease had confusion at the time of hospital admission, whereas only 10 (7.4 percent) patients were documented to have CNS infection [11]. Given the aim of the study that was the evaluation of the etiologic distribution of acute fever in the elderly, small number of aged adults with fever and confusion were included.

In this part, we try to estimate the frequency of CNS infection among AFE elderly patients.

*Systematic Review*. A thorough search in the literature revealed only that few studies provided data about the frequency of CNS infection in elderly patients with AFE to be included (Table 1). We excluded articles in which the field of interest was restricted to a few specific pathogens and articles that only investigate the etiologic diagnosis of BM in elderly patients. Finally, we found only 4 studies performed to address this issue. All, except one that did not mention the design of study, used a retrospective design. The results showed the frequency of 2.4 to 24 percent for CNS infection among AFE elderly patients. However, the small sample size, retrospective design, and selection bias of these studies that only included patients who underwent lumbar puncture (LP) make an estimation of true prevalence of CNS infection in aged adults with AFE impossible.

#### 2.2.3. Third Question: Does Sepsis-Associated Encephalopathy (SAE) Explain the Higher Prevalence of Acute Febrile Encephalopathy (AFE) among the Elderly That Require Hospitalization?

The incidence of sepsis among individuals 85 years of age and older has been estimated to be 26.2 cases per 1000 populations, which is more than 100 times greater than the incidence noted among individuals 5 to 14 years of age [33]. According to Marco et al. the most common final diagnoses among geriatric emergency department (ED) patients who presented with acute fever were pneumonia, urinary tract infection, and sepsis [34].

Among the myriad of conditions that can induce delirium in critical illness, sepsis in the form of SAE represents the most frequent and severe cause [35]. Up to 70 percent of patients with bacteremia have wide spectrum of neurological symptoms that include fluctuating mental status changes, inattention, and disorganized thinking and therefore match with current criteria for delirium [36, 37]. The cardinal feature of SAE is a diffuse disturbance in cerebral function without any lateralizing signs. Two key prerequisites for making a diagnosis of SAE are presence of extracranial infection and impaired mental status. Diagnosis of brain dysfunction in a patient with sepsis implies a systematic diagnostic approach of all potential factors, in addition to sepsis, that can contribute to aggravate or prolong brain dysfunction [35]. Nonspecific clinical expressions of infection are common in elderly patients. In addition to the frequent lack of fever, infections in older adults may be associated with a nonspecific decline in baseline functional status such as increased confusion and falling. Cognitive impairment further contributes to the atypical presentation of infections in older adults, reducing the capacity to communicate symptoms [12].

Severe sepsis in older population is associated with substantial and persistent new cognitive impairment and functional disability among survivors. The magnitude of these new deficits is large, likely resulting in a pivotal downturn in patients' ability to live independently. In fact, sepsis is often a sentinel event in the lives of older patients, initiating major and enduring cognitive and functional declines. Iwashyna et al. studied the cognitive impairment of older patients with severe sepsis (1194 patients, mean age of survivors 76.9 years) and found that prevalence of moderate to severe cognitive impairment increases by 10.6 percentage points among patients who survived severe sepsis [38].

Although older age is a known risk factor for SAE, it does not seem to* fully* explain the frequent presentation of acutely ill febrile older adults with encephalopathy syndrome. On the other hand, it has been noted that SAE is a diagnosis of exclusion: there should be no clinical or laboratory evidence of direct central nervous system (CNS) infection (e.g., meningitis, macroscopic intracranial abscess, or empyema), head trauma, fat embolism, adverse reactions to medications, or sedative or paralyzing drug effects [36]. It has been suggested that occurrence of sudden fluctuation in mental status, occurrence of focal neurological sign, seizure(s), and/or neck stiffness should prompt the physician to consider neuroimaging, electroencephalogram (EEG), and/or lumbar puncture (LP) to rule out a direct CNS infection [35]. It has been recommended to perform a LP on obtunded patients with systemic inflammatory response syndrome (SIRS), to rule out meningitis [36]. Considering the high frequency of altered mental status in elderly patients with infectious syndromes outside the CNS, is it reasonable to perform early LP on every confused aged adult with fever or sepsis syndrome? Answering to this question requires the knowledge about the frequency of CNS infection and SAE in elderly patients with AFE.

*Systematic Review*. On the basis of literature review, we could not find any relevant prospective study to the frequency of SAE in elderly patients with AFE. However, the role of older age as a risk factor for SAE has been investigated in several studies [38]. In few retrospective studies, the frequency of abnormal CSF in elderly patients with AFE varied between 2.4 to 24 percent (Table 1). Nonetheless, because of the retrospective design of these studies it cannot be interpreted as an estimation of SAE in elderly patients with AFE.

#### 2.2.4. Forth Question: Is the Classic Triad of Meningitis, a Sensitive and Specific Indicator of Central Nervous System (CNS) Infection among Elderly Patients? Is the Combination of Nuchal Rigidity and Encephalopathy a Specific Indicator of CNS Infection in Elderly Patients with Acute Febrile Encephalopathy (AFE)?

Early recognition of acute bacterial meningitis (ABM) is challenging. Common clinical practice relies on the absence of neck stiffness or other meningeal signs to rule out meningitis in the previously healthy adult. Meningeal signs have been assumed to be reliable and usually present in awaked adults with meningitis, except infants, the elderly, and the immunesuppressed [39]. According to van de Beek et al. in adults presenting with community-acquired bacterial meningitis (BM), the sensitivity of the classic triad of fever, neck stiffness, and altered mental status is low (44 percent), but almost all (95 percent) present with at least two of the four symptoms of headache, fever, neck stiffness, and altered mental status [40]. It has been noted that among adults with a clinical presentation that is low risk for meningitis, the clinical examination aids in excluding the diagnosis [41]. Nevertheless, in the study of Waghdhare et al. physical signs of meningeal inflammation were not helpful for ruling in or ruling out meningitis accurately (age range of participants: 13–81; mean: 38 ± 18) [22] Similarly, Brouwer et al. reported low diagnostic accuracy of signs of meningeal irritation for prediction of cerebrospinal fluid (CSF) pleocytosis, suggesting that clinical assessment alone is insufficient to exclude BM [42]. In addition, Stockdale et al. found that, in patients with ABM, the classical clinical features are uncommon on arrival to hospital and frequently evolve following admission [43]. Waghdhare et al. recommended patients suspected to have meningitis to undergo a LP regardless of the presence or absence of physical signs [22].

BM in elderly patients is associated with greater diagnostic difficulties and more complications, as well as with increased mortality [44]. According to Choi in the clinical assessment for possible BM, older adults who present without fever, neck stiffness, or altered mental function probably do not have this disease. Those individuals with 2 or 3 of the 3 classic findings are more likely to have meningitis, but even the presence of all 3 findings is not entirely specific [29]. Since febrile responses are often blunted or absent in older adults, it is not surprising that fever is not a universal symptom, varying in occurrence from 59 to 100 percent in different studies. Similarly, headache has been noted in only some older adults with meningitis, and depressed levels of consciousness such as stupor or coma are often but not universally present [29, 45]. Diagnostic difficulty of ABM in the elderly is attributed to its atypical and more subtle presentation [10, 46]. It has been shown that the time from arrival to starting antibiotic is longer among those patients with suspected meningitis who had atypical presentation or complex medical histories [47]. Another major problem in attaining a correct diagnosis in the elderly is the presence of multiple pathologies. Although the majority of elderly patients present with fever, confusion, and stiff neck, confusion may be assumed to be secondary to senility, and the stiff neck to cervical osteoarthritis. In such circumstances one would recommend more frequent “spinal tap” as a prerequisite to exclude meningitis in the elderly [48]. It seems that nuchal rigidity is neither sensitive nor specific sign compared with younger patients [45]. Nuchal rigidity is often found on examination of elderly patients who do not have BM and usually have coexistent neurologic deficits. The issue to be discussed here is how one can be sure if the neck stiffness or rigidity in an elderly patient is of new onset.

According to the study performed by Puxty et al. nuchal rigidity, which may be a sign of meningitis, was found in 35 percent of geriatric patients on acute-care and rehabilitation wards and was significantly associated with cerebrovascular disease (CVA), confusion, abnormal plantar responses, and primitive reflexes. Accordingly, they suggested that elderly patients who have nuchal rigidity with no history of neurologic or cognitive disorders should be investigated for meningitis [49].

Neck stiffness in older adults without meningitis may be caused by prior CVA, cervical osteoarthritis, Parkinson's diseases, or certain drugs [29]. Considering the high prevalence of these underlying conditions among the elderly, interpretation of clinical* tests* evoking* meningeal irritation* may be inconclusive in significant number of hospitalized aged adults. For example, it has been estimated that overall prevalence of parkinsonism is more than 15 percent for people 65 years and older and its prevalence increases markedly with age. The prevalence of mild parkinsonian signs is even higher and has been reported to exceed 30 percent among community-dwelling older people [50, 51]. Although it has been suggested that hypotonicity of the neck muscle resulting from diseases of basal ganglia, such as parkinsonism, can be distinguished from true nuchal rigidity, in practice this differentiation is difficult [52]. Resistance to passive movement of the neck is a common physical finding in elderly patients because of the presence of cervical spondylosis. It has been recommended in the reference books that elderly patients who have nuchal rigidity in the absence of other neurologic problems should not be dismissed as having “osteoarthritis of the cervical spine” but should be intensively investigated for possible meningitis [45]. Although it is practically difficult to distinguish between the cervical spondylosis and nuchal rigidity resulting from meningitis, some clinical clues have been proposed. For example, it has been noted that, in nuchal rigidity, the neck resists flexion but in spinal disease, lateral rotation, extension, and flexion of the neck are all associated with resistance. It has also been suggested that a somewhat helpful clinical sign is that with cervical osteoarthritis, in particular, passive flexion of the neck may elicit resistance more at the extremes of range of motion, whereas with meningeal irritation, resistance may be felt more immediately [29]. Not only test of passive flexion of the neck is unreliable in elderly patients, but also Kernig and Brudzinski signs are probably of little or no diagnostic value [53].

Although it has been reported that more than 80 percent of elderly patients with BM have nuchal rigidity [54, 55], several other studies have noted that the diagnosis of BM is more difficult in the elderly because of the absence of characteristic meningeal signs [44]. In a series of CNS infections in aged patients, only 57 percent of 28 patients had meningismus [56]. Weisfelt et al. found that elderly patients were less likely to have neck stiffness than younger adults but more likely to have impairment of consciousness [23]. Rasmussen et al. showed that the most common symptoms of BM in elderly patients were fever 79 percent, change in mental status 69 percent, and meningismus 54 percent [24]. According to the study of Domingo et al., elderly patients had comorbid conditions more frequently and more frequently lacked fever and neck stiffness but had an altered level of consciousness more often [31]. In a review that compared the clinical presentation of BM in elderly patients with that in younger patients, the incidence of more severe abnormalities of mental status in the two groups was significantly different. In this study that included 54 cases of BM in the elderly, confusion was presented in 92 percent of the patients with pneumococcal meningitis and in 78 percent of those with Gram-negative meningitis on initial presentation [55]. Data from studies addressing the nontraumatic, spontaneous Gram-negative bacillary meningitis in the elderly or debilitated patients showed that the classic signs and symptoms of meningitis may be subtle at initial presentation. These are assumed as a distinct group of elderly patients who may have only low-grade fever and altered mental status without headache or nuchal rigidity; however, patients with spontaneous Gram-negative bacillary meningitis tend to have a rapidly progressive fulminant course associated with bacteremia, shock, and coma after presenting what at first appeared to be a minor illness [52].

The contemporary recommendation is that meningitis should be suspected in every elderly patient who is febrile and either disoriented, stuporous, or comatose. But what is the true prevalence of CNS infection among these patients? Is encephalopathy a specific sign for CNS infection in elderly patients? When febrile responses and systemic inflammatory syndromes are often blunted in older adults, is it necessary to manage any elderly patient with acute alteration in mental status as the patient with CNS infection?

*Systematic Review*. On the basis of literature review, we could not find any prospective study about the frequency of meningeal signs in elderly patients who present with AFE and their relations to the etiologic diagnosis. The frequency of various signs and symptoms in AFE elderly patients varies among different studies on the basis of the design of the studies, as well as the variables studied. According to the few studies in elderly patients (Table 2), meningeal signs are not universal finding in older adults, varying in occurrence from 54 to 82 percent in different studies. However, most of them were designed retrospectively. It seems that meningeal signs are insensitive and nonspecific for CNS infection among elderly patients. Most elderly patients with meningitis present only with fever and altered mental status. Even when clinical findings suggestive of neck stiffness or other meningeal signs existed in an older patient, the high frequency of underlying diseases such as cervical spondylosis and Parkinson's disease makes these findings difficult to interpret. Comparing with adult patients younger than 65 years of age, the older patients present more often (up to one third) with neurologic deficits and show greater neurologic severity with a high number presenting with coma on admission, seizures, and hemiparesis [44, 55].

#### 2.2.5. Fifth Question: Is a Lumbar Puncture (LP) Necessary When Evaluating an Older Patient with Acute Febrile Encephalopathy (AFE)? How Much Elderly Patients with AFE Undergo LP: Too Many or Too Few?

Early recognition and treatment of acute community-acquired bacterial meningitis (BM) are essential to improve the prognosis of the disease. Although textbooks of infectious diseases recommend appropriate antibiotic administration within 30 minutes of presentation, recent studies consistently report substantial delays in the time to antibiotic administration, ranging from 2 to 4.9 hours [57]. There is an independent incremental association between delays in administrating antibiotics and mortality from adult acute bacterial meningitis (ABM). Inappropriate diagnostic-treatment sequences are significant predictors of such treatment delays [57]. Because of the excessive morbidity and mortality associated with delays in treatment of BM and insufficiency of clinical assessment to exclude central nervous system (CNS) infection, it is common practice to perform an immediate LP upon any patient with suggestive symptoms, no matter how unlikely the diagnosis is thought to be. Statements such as “If you think of doing a spinal tap, do one” are seen in standard medical textbooks [58].

As mentioned before, nonclassical presentations of acute illnesses occur frequently in the frail elderly and acute infections including CNS infections are no exception [6]. None of us is accurate enough with a physical exam to reliably determine if a febrile elderly patient with confusion has an underlying CNS infection or not. More than 50 percent of all deaths from meningitis occur in persons aged 60 and older. Several reasons have been proposed to account for this observation. First, elderly patients with meningitis more often develop complications than younger adults, which resulted in a higher mortality rate [10, 59]. According to Weisfelt et al. older people with meningitis tended to die more often from cardiorespiratory failure, whereas younger adults more often died from brain herniation. Although they attributed the poorer outcome of the elderly to higher rate of pneumococcal meningitis in this age group, multivariate analysis revealed that older age is an independent risk factor for adverse outcome even after adjustment for the causative pathogen [23]. The second major reason for high mortality rate of meningitis in older patients is delay in diagnosis. In fact, because of the nonspecific presentation of CNS infection among older adults, it is likely that a significant proportion of these infections in the elderly goes unrecognized and untreated especially in the presence of stroke or dementia [48]. On the other hand, there are also some findings which appear to suggest that the examination of CSF in the emergency department (ED) may not always be necessary and that some cerebrospinal fluid (CSF) tests may be excessive [26].

The standard care is that once there is suspicion for ABM, blood cultures must be obtained and a LP is performed immediately to determine whether the CSF findings are consistent with the clinical diagnosis. In the circumstances in which the clinician cannot emergently perform the diagnostic LP or is concerned that the clinical presentation is consistent with a CNS mass lesion or another cause of increased intracranial pressure and wants to obtain a computed tomography (CT) scan of the head prior to LP, blood cultures must be obtained and appropriate antimicrobial and adjunctive therapy given to the patient prior to LP [60].

LP is frequently performed in the ED, mostly for suspicion of CNS infection, which is eventually confirmed in one-third of cases [61]. In the study of Powers charts of 104 adult patients who underwent LP in a university hospital ED were reviewed. Examination of the CSF revealed pleocytosis in 24 percent of the patients. According to the preponderance of negative or normal results of CSF analysis, authors suggested that extensive testing may not be necessary for all patients [62]. On the other hand, in the study conducted by Khasawneh et al. the results of LP led to a change in management in 30 percent of critically ill medical patients who admitted to the intensive care unit (ICU) [63].

Many experts recommend that all older adults with acute onset of fever and confusion should be treated with empiric antibiotics for CNS infection including BM and herpetic encephalitis and have LP performed after neuroimaging in the first hours after admission. Nevertheless, it has also been suggested that because BM is an uncommon disorder in elderly patients, routine CSF evaluation may not be necessary in all febrile or septic appearing older patients with delirium as long as other infectious foci are obvious. Given the lack of specificity or sensitivity of symptoms and signs in the elderly, the basis for the diagnosis of meningitis is the LP, with analysis of the CSF [29]. Chakravarty et al. suggested that CSF should be analyzed in atypical cases of stroke, or when pyrexia develops without an apparent source of infection in an elderly patient with stroke [48]. However, this is not seemed to be the common practice. Although for other age groups, a major distinction must often be made between bacterial and viral meningitis, more common clinical problem in the geriatric population is distinguishing between BM and infection at another site as the cause of fever and acute alteration of mental function [29]. Considering published literature data, only few surveys studied the use and the diagnostic efficiency of LP in AFE adults. LP efficiency is assumed to be modest especially among elderly patients when an infection of the CNS is suspected [26]. The question to be answered is whether it is reasonable to recommend early LP as part of routine diagnostic workup of every AFE elderly patient who admitted to ED after normal neuroimaging results.

According to Choi in deciding whether LP is indicated, one must consider the pretest likelihood of meningitis, and since this is a complex clinical analysis, it is difficult to provide a rigid guideline. They noted that some patients with fever, acutely depressed mental function, and infection at a nonmeningeal site may be treated for the infection and closely observed without LP; however, most patients who develop these symptoms should probably undergo LP if it is safe to do so, particularly if their symptoms began before hospitalization [29]. We try to make a conclusion for determining the threshold of performing LP in elderly patients with AFE. Making a comparison between neonates and elderly populations could help us understand the problem. The young febrile infant may demonstrate few, if any, interpretable clues to the underlying illness. The limitations of the history and physical examination in neonates and young infants with fever traditionally have led to an aggressive laboratory evaluation, even for patients who were previously healthy, are well-appearing, and have no focal infection [64]. To some extent this is the same for significant number of febrile elderly who are admitted to ED especially those with AFE. In the past, it was the standard of care that most young febrile infants including all febrile neonates 28 days of age or younger have blood, urine, and CSF cultures performed regardless of clinical appearance. These infants had been admitted to the hospital for antibiotic treatment pending negative cultures. Subsequently, criteria have been developed that can identify young infants with fever who are at low risk for serious bacterial illness and can be safely managed as outpatients. However, the available guidelines and approaches to fever in young infants still recommend that all febrile neonates 28 days of age or younger have sepsis workup including LP and treated with empiric antibiotics regardless of clinical appearance [64]. The underlying basis for this recommendation is the relatively high prevalence of serious bacterial infection and high frequency of BM in bacteremic neonates. BM occurs in as many as 15 percent of neonates with bacteremia [65]. Let us make a comparison: If the frequency of CNS infection among elderly patients with AFE was high, it would be reasonable to perform early LP for every febrile aged patient with acute alteration in mental status after neuroimaging. In contrast, if the frequency was considerably low, this management would cause unacceptable aggressive diagnostic evaluation and cost.

*Systematic Review*. Unfortunately, the systematic review for making such a conclusion revealed few retrospective studies. We found only 4 studies that evaluated the efficiency of LP in old aged adult patients with AFE.

*Study Number 1.* In the study conducted by D'Amore and Nelson among 191 elderly patients presenting with fever and altered mental status who underwent LP, 21 percent of patients had a source identified by CSF analysis: 10 (6.2 percent) cases of BM, 21 (13 percent) cases of viral meningitis, and 4 (2.5 percent) cases of viral encephalitis. The authors of the study concluded that patients with fever and altered mental status and a pre-LP source of infection may have a higher rate (9.5 versus 2.8 percent) of BM. The results of the study showed that when a pre-LP source is found, nursing home patients appear less likely to receive LP. Despite the retrospective design of the study and inclusion of only those patients who underwent LP, they suggested that ED physicians should perform LP on all elderly patients with fever and altered mental status [19].

*Study Number 2.* Shah et al. conducted a retrospective study to compare the diagnostic utility of LP in febrile versus afebrile elderly patients with altered mental status. Their null hypothesis was that there is no utility of performing an LP on the afebrile delirious elderly patients. Of 125 patients, 84 were afebrile and 41 had fever. Eighteen percent of afebrile patients and 24 percent of 41 febrile patients had abnormal CSF. Comparing the elderly patient group without fever with the elderly patient group with fever, they could not reject their null hypothesis and suggested to not relying solely on the presence or absence of fever to determine management in the elderly [20].

*Study Number 3.* Alavi and Moogahi studied 60 elderly patients with confusion and fever in a teaching hospital to determine the causes of confusion and fever and identify the necessity of the CSF examination. Of the total patients in this study, 10 percent were diagnosed as BM. The remaining were diagnosed as bacteremia. The results of the study showed that older age was not a statistically significant predictor of BM in patients with acute onset of fever and confusion. They also found that among elderly persons, extra-meningeal infection with or without underlying illness is a more important cause of confusion and fever than BM. They concluded that elderly patients with fever and confusion, without signs of meningeal irritation, may not require a routine LP performance for evaluation of their CSF [21].

*Study Number 4*. Warshaw and Tanzer reviewed 81 hospitalized elderly patients who underwent LP. Seventy percent of LPs were performed as part of the admitting workup, and the remaining 30 percent during the hospitalization. They found only one case of BM and the authors concluded that most hospitalized elderly patients with febrile delirium have primary causes of the confusion outside the CNS and may not require a routine CSF analysis [18].

In the retrospective study conducted by Majed et al. with the aim of evaluating the frequency of use and the diagnostic efficiency of LP, total of 247 patients, representing 0.5 percent of all ED admissions, underwent a LP. The main assumed LP indications were to search for CNS infection 62 percent and for subarachnoid hemorrhage (SAH) 25 percent. LP was efficient in fewer than 15 percent of cases and confirmed aseptic meningitis 8.5 percent, BM 2.4 percent, Guillain-Barré syndromes 1.6 percent, SAH 0.4 percent, and carcinomatous meningitis 0.4 percent. The principal differential diagnoses were infections outside the CNS, noninfectious neurological disorders, and benign headaches. LP efficiency decreased dramatically according to patients' age. Accordingly, contributive LP represented 13.4 percent (95 percent CI: 9.1–17.6) of the cases and varied significantly according to patients' age: the proportion of efficient LP decreased from 25 percent among young patients to 14.2 percent among middle-aged patients and to less than 5 percent among elderly patients. In this study, total number of elderly patients who underwent LP with the suspicion of CNS infection was 87 of whom 55 (82 percent) cases had confusion and 36 (53.7 percent) were febrile. Of the total elderly patients 43.7 percent had infection outside the CNS and only 3.4 percent had meningitis. In 4 (4.6 percent) elderly patients, LP was contributive [26].

With regard to answering the question of this part, one should estimate the missing number of CNS infections in the elderly patients who present to ED including those who die undiagnosed. Because of the retrospective design of most studies about CNS infection in elderly patients, the estimation is impossible. On the other hand, nearly all studies with regard to this topic are limited by selection bias of only those patients who undergo LP. Maybe, studies based on postmortem examinations would be able to make this estimation.

A number of the studies described in this section seem to point out that many elderly patients with AFE do not require LP. However, because of the insufficient number of the studies performed to address the issue, their heterogeneity, and retrospective design, drawing a conclusion about the proper threshold for performing LP in elderly patients with AFE is somewhat difficult.

## 3. The Necessity of Developing an Evidence-Based Guideline

Although clinical judgment of individual patients by their physicians remains the most important factor in the diagnosis of acute bacterial meningitis (ABM) [42], the use of evidence-based guidelines help standardize care among physicians, as well as various institutions [64]. Prognostic classification of disease remains a powerful tool for the bedside clinician in diagnostic and management decision making. Accurate and valid prognostic models are difficult to develop, because they require detailed clinical data collection from a large cohort of patients with a clinically relevant outcome assessment. For some of the most common infectious diseases, prognostic classification with validated scoring systems is used. However, because of including heterogeneous adult population and their primary outcomes that are limited to the mortality end-points instead of more relevant outcomes for the elderly such as rate and duration of functional decline, their applicability to the care of older adults is limited [42, 66]. Although the few existing prediction models can be used to estimate the risk of ABM, these models need to be refined and validated further in specific settings and populations such as the elderly. Refining and validating prediction models for a specific age group could be helpful in developing practical diagnostic and therapeutic recommendations and evidence-based guidelines in the age group. In this regard, we suggest conducting further prospective well-designed studies with adequate sample size that focus on the following aims:

1. to estimate the true prevalence of central nervous system (CNS) infection among elderly patients who present with acute febrile encephalopathy (AFE),
2. to define different set of classic clinical criteria for meningitis in elderly patients (instead of considering their presentation “atypical” most of the time),
3. to determine the proper threshold for performing lumbar puncture (LP) in elderly patients who present with AFE,
4. to design a practical evidence-based guideline to help physicians to recognize elderly patients who benefit from early LP, those for whom delaying LP is not harmful, and those who do not require LP at all.

## 4. Conclusion

Unfortunately, our attempt is to develop a recommendation about the optimal threshold of lumbar puncture (LP) defeated because of the inadequate number and weak evidence from retrospective studies performed to address the issue in the literature. Considering the diagnostic dilemmas in elderly patients with acute febrile encephalopathy (AFE), it would be more useful to design guidelines around this topic based on a clinical syndrome, that is, AFE not based on disease category, that is, bacterial meningitis (BM). In addition, prospective studies with the aim of revising or validating clinical predictors of central nervous system (CNS) infection in elderly patients are welcomed. Accordingly, it is mandatory to perform prospective well-designed studies with the aim of evaluating the etiologic diagnosis of AFE in elderly patients and the proper sequence of diagnostic and therapeutic management.

## Figures and Tables

**Table 1:** Summary of the analyzed studies about the frequency of CNS infection in elderly patients with AFE.

	Warshaw and Tanzer [18]	D'Amore and Nelson [19]	Shah et al. [20]	Alavi and Moogahi [21]
Number of participants	81	191	125	60
Number of elderly with AFE	81	191	41	60
Study design	Retrospective	Retrospective	Retrospective	Not mentioned
Location	Cincinnati, Ohio. Single center	Manhasset, NY. Multicenter	New York, USA. Single center	Ahwaz, Iran. Single center
Primary end point	To determine the value of the CSF examination	To analyze the contribution of LP	To determine diagnostic utility of LP	To determine the necessity of LP
Inclusion criteria	Elderly patients with fever and delirium	Elderly patients with fever and altered mental status	Febrile and afebrile elderly patients with altered mental status	Elderly patients with confusion and fever
Frequency of CNS infections among participants	2 (2/4%)	35 (21%)	20%	6 (10%)
Frequency of CNS infection among elderly with AFE	2 (2/4%)	35 (21%)	10 (24%)	6 (10%)

AFE: acute febrile encephalopathy; LP: lumbar puncture; CSF: cerebrospinal fluid; CNS: central nervous system.

**Table 2:** Studies in the literature performed to address the meningeal signs in adults (only studies included that at least make an estimation of the numbers of older people).

	Waghdhare et al.^*∗*^ [22]	Weisfelt et al. [23]	Rasmussen et al. [24]	Thomas et al.^*∗*^ [25]	Alavi and Moogahi [21]	Majed et al. [26]
Location	Maharashtra, India	Netherlands	Denmark	New Haven, USA	Ahwaz, Iran	Arras, France

Date	2010	1998–2002	1976–1988	2002	2006-2007	2004-2005

Age	13–81 (38 ± 18)	Elderly: 71 ± 7.5 Younger: 38 ± 14		18–93 (40)	Male: 74.4 ± 6.21 Female: 73.8 ± 8.31	36–72 (52)

Inclusion criteria	Acute encephalitis syndrome (≥12 yrs)	Culture proven community-acquired bacterial meningitis (≥16 yrs)	Acute bacterial meningitis (≥60 yrs)	Adults with suspected meningitis	Fever and confusion (≥56 yrs)	All adult patients who underwent an LP

Study Design	Double blind, cross-sectional	Prospective, cohort	Retrospective	Prospective	Not mentioned	Retrospective

Total number of participants	190	696	48	297 Number of cases with meningitis: 80	60 Number of cases with meningitis: 6	247

Total number of elderly patients	25/190 (13.1%)	257/696 (37%)	48/48 (100%)	46/297 (15.8%) Number of elderly patients with meningitis: 9/80 (11.2%)	60/60 (100%) Number of elderly patients with meningitis: 6/6 (100%)	87/247 (35.2%) Number of elderly patients with meningitis: 3/87 (3.4%)

Results	Nuchal rigidity: LR+ 1.33 (0.89, 1.98) LR− 0.86 (0.70, 1.06) Kernig: LR+ 1.84 (0.77, 4.35) LR− 0.93 (0.84, 1.03) Brudzinski: LR+ 1.69 (0.65, 4.37) LR− 0.95 (0.87, 1.04) Jolt: LR+ 5.52 (0.67, 44.9) LR− 0.95 (0.89, 1.00)	Neck stiffness: elderly 78% versus younger 81% Classic triad (fever, neck stiffness, and altered mental status): Elderly 58% versus younger 36%	Meningeal signs: 54%	Nuchal rigidity: sensitivity 30%, LR+ 0.94 Kernig: sensitivity 5%, LR+ 0.97 Brudzinski: sensitivity 5%, LR+ 0.97	Meningeal signs: 6/6 (100%) of bacterial meningitis versus 32/54 (59%) of elderly patients with fever and confusion who had infection outside the CNS	Neck stiffness: elderly patients who underwent LP: 31/87 (35%) of whom only 3/87 (3.4%) had meningitis 33.9% of patients less than 35 years and 29.8% of patients between 35 and 65 years had neck stiffness, of whom 23.2% and 11.5% had meningitis

^*∗*^No comparison was made between elderly and younger patients in regard to the frequency of meningeal signs

LP: lumbar puncture; LR: likelihood ratio.
